# Examination of Influenza Specific T Cell Responses after Influenza Virus Challenge in Individuals Vaccinated with MVA-NP+M1 Vaccine

**DOI:** 10.1371/journal.pone.0062778

**Published:** 2013-05-03

**Authors:** Timothy J. Powell, Yanchun Peng, Tamara K. Berthoud, Marie-Eve Blais, Patrick J. Lillie, Adrian V. S. Hill, Sarah L. Rowland-Jones, Andrew J. McMichael, Sarah C. Gilbert, Tao Dong

**Affiliations:** 1 MRC Human Immunology Unit, Weatherall Institute of Molecular Medicine, University of Oxford, John Radcliffe Hospital, Oxford, United Kingdom; 2 Jenner Institute, University of Oxford, Oxford, United Kingdom; The University of Adelaide, Australia

## Abstract

Current influenza vaccines stimulate neutralising antibody to the haemagglutinin antigen but as there is antigenic drift in HA it is difficult to prepare a vaccine in advance against an emergent strain. A potential strategy is to induce CD8^+^ and CD4^+^ T cells that recognize epitopes within internal proteins that are less subject to antigenic drift. Augmenting humoral responses to HA with T cell responses to more conserved antigens may result in a more broadly protective vaccine. In this study, we evaluate the quality of influenza specific T cell responses in a clinical trial using MVA-NP+M1 vaccination followed by influenza virus challenge. In vaccinated volunteers, the expression of Granzyme A, Perforin and CD57 on influenza HLA A*02 M1_58–66_ antigen specific cells was higher than non-vaccinated volunteers before and after challenge despite a similar frequency of antigen specific cells. BCL_2_ expression was lower in vaccinated volunteers. These data indicate that antigen specific T cells are a useful additional measure for use in human vaccination or immunization studies.

## Introduction

Protection against influenza virus requires antibody secretion by B cells and cytotoxic and soluble mechanisms mediated by T cells [1]. The antibody response can be stimulated by vaccination and the existence of an influenza specific haemaggutination-inhibition antibody titre of 1∶40 or more is associated with protection [2]. Influenza virus undergoes antigenic shift and drift, generating novel influenza viruses to which people may not have immunity [3]. One way of overcoming this lack of immunity could be to stimulate pre-existing cross-reactive CD4^+^ and CD8^+^ T cell responses which have been shown in humans to react with H1N1 2009 virus [4] and H5N1 [5], [6]. This heterosubtypic immunity is associated with protection during human experimental influenza infection [7]. CD8^+^ T cell responses to one conserved A*02 matrix protein 1 (M1)_58–66_ epitope can be protective in A2 transgenic mouse models [8] and are commonly found in healthy donors with this common HLA type [9]. Therefore it is convenient to analyse M1_58–66_ specific CD8^+^ T cells in vaccine studies. The phenotype or activation state of T cells is important for protection against influenza such that naïve cells are less able to protect than activated or memory cells against a lethal influenza infection [10], [11]. Activated influenza specific T cells have been shown to be associated with protection against influenza in human studies [12], [13], [14], [15] but no study of surface or intracellular phenotype was done.

We sought to characterize the antigen specific immune response to influenza following vaccination with a viral-vectored nucleoprotein+matrix protein 1 (NP+M1) influenza vaccine and subsequent influenza challenge and determine whether there was any change in the phenotype and functional potential of antigen specific CD8^+^ T cells. We found that following vaccination with modified vaccinia Ankara (MVA)-NP+M1, antigen specific M1_58–66_ CD8^+^ T cells showed a more enhanced activation profile showing higher levels of perforin, granzyme A and CD57. There was also a reduction in BCL_2_ expression. These antigen specific cells expanded in response to challenge with live influenza virus. The vaccine-stimulated cells were altered in terms of their surface and intracellular phenotype. Examination of the phenotype of antigen specific T cells may be a useful adjunct for human immunization studies.

## Materials and Methods

### Ethical Permissions and Human Studies

Volunteers were recruited using an approved Medicines and Healthcare products Regulatory Agency and the Oxfordshire Research Ethics committee protocol, and enrolled only after obtaining written informed consent (www.clinicaltrials.gov identifier: NCT00993083, approved 19 May 2009). Volunteers aged 18–45 were recruited at the Centre for Clinical Vaccinology and Tropical Medicine, Oxford and the Welcome Trust Clinical Research Facility, Southampton beginning 08 Jun 2009. Volunteers were initially screened by haemagluttination inhibition assay against the virus to be used in the influenza challenge phase of the study. Those with a titre ≤1∶10 were eligible for further screening. Volunteers were seronegative for HIV, Hepatitis B virus and Hepatitis C virus and had not received seasonal influenza vaccination for at least one year prior to enrolment. Routine haematological and biochemical tests on enrolled volunteers were all within normal limits [16].

### Vaccine, Vaccinations and Virus Challenge

**Figure 1:**
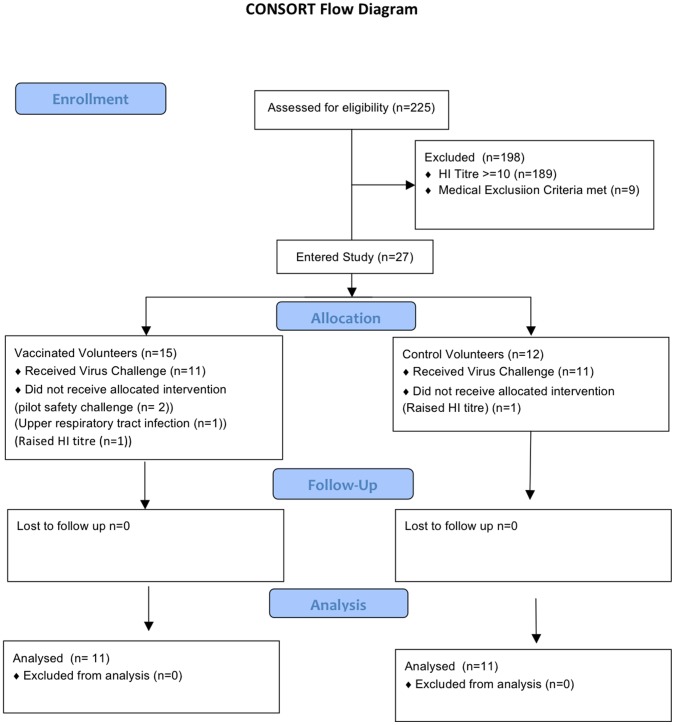
CONSORT flow diagram of the clinical trial.

The CONSORT flow chart for the trial is shown in figure 1. Beginning 27 July 2009, an MVA vaccine [17] expressing influenza (H3N2 A/Panama/2007/99) NP+M1 was administered, 1.5×10^8^ plaque-forming units (PFUs), to 11 human volunteers 1 month before challenge with influenza (H3N2 A/Wisconsin/67/2005). Control subjects were challenged with H3N2 virus only. The viral challenge study was conducted by Retroscreen Virology Ltd [16]. Symptoms and virus shedding were monitored. Differences between IFN-γ ELISPOT analysis pre and post challenge are given in [16]. To examine the acute response after virus infection, blood was taken one day before (−1) and days 4 and 7 after challenge, transported to Oxford and then flow cytometry performed on whole blood.

### Flow Cytometry Analysis of Whole Blood Samples

Whole blood was aliquoted into tubes and then labelled with six different antibody panels all with tetramer-PE: HLA-A*0201 complexed with M1_58–66_ peptide GILGFVFTL, produced in house using standard methods [18] and incubated for 15 mins at 37°C. Red blood cells were lysed using RBC lysis media (Becton Dickinson, Oxford UK) for 15 mins at room temperature then washed 2× with FACS buffer PBS (Difco, Poole) with 1% v/v BSA (Sigma, Poole UK) followed by incubation with CD8-PerCP and CD4-Pac Blue (eBiosciences, Hatfield UK) plus different panels of ab: CD28-FITC. HLA-DR-APC, CD38-PE-Cy7 and CD27-APCH7 (eBiosciences) or CD57-FITC and CD25-APC or CD45RA-FITC, CD45RO-PECy7 and CCR5-APC. Cells allocated to the Intracellular panels were permeabilised with Perm2 (BD) for 15 mins and washed 2× in FACS buffer. Cells were then labeled with CD8-PerCP (Biolegend) and CD4-Pac Blue followed by: Perforin-FITC (D48, Genprobe, Manchester, UK) or GranzymeA-FITC or GranzymeB-FITC or Ki67-FITC. Cells were then washed twice and fixed in BD cellfix. All abs were from Becton Dickinson (Oxford, UK) unless otherwise stated. Similar staining protocols were also done using the CMV lower matrix protein pp65_495–503_ NLVPMVATV tetramer [19]. Cell events were collected on a 9 colour Cyan Cytometer (Dako, Ely, UK) and data files analysed using FlowJo (Tree Star Inc, Ashland, OR, USA). Data were analysed using a forward side scatter gate followed by CD8 gating then tetramer gating within the CD8+ population. These cells were then analysed for percentage expression of a particular marker using unstained and CD8+ tet- populations to determine where to place the gates. Single colour samples were run for compensation and fluorescence minus one (FMO) samples were also run to check positive and negative populations as well as channel spillover. To monitor overall changes of white blood cell numbers, whole blood samples were analysed using the BD Trucount system measuring CD4, CD8 and CD3 positive lymphocytes according to manufacturers instructions.

### ELISPOT Analysis of PBMC

PBMC were separated on density gradients and incubated with peptide pools from each gene of the H3N2 virus along with peptide pools from other subtypes of haemagglutinin (HA) and neuraminidase (NA) as described previously [16]. Peptide pools were similar to those described previously [20]. Swine origin (SO) HA and NA were overlapping 18–20 mers from the full sequence of A/Cal/04/2009 H1N1 influenza virus.

### Statistical Analyses

Groups of data were analysed by repeated measures ANOVA using pairing of samples using the statistical package R (R Foundation). Changes between vaccinated and control were considered along with time and interaction between both. Any analyses showing p values <0.05 were considered significant. Data from repeated measures ANOVA are shown in Table S1.

## Results and Discussion

**Figure 2:**
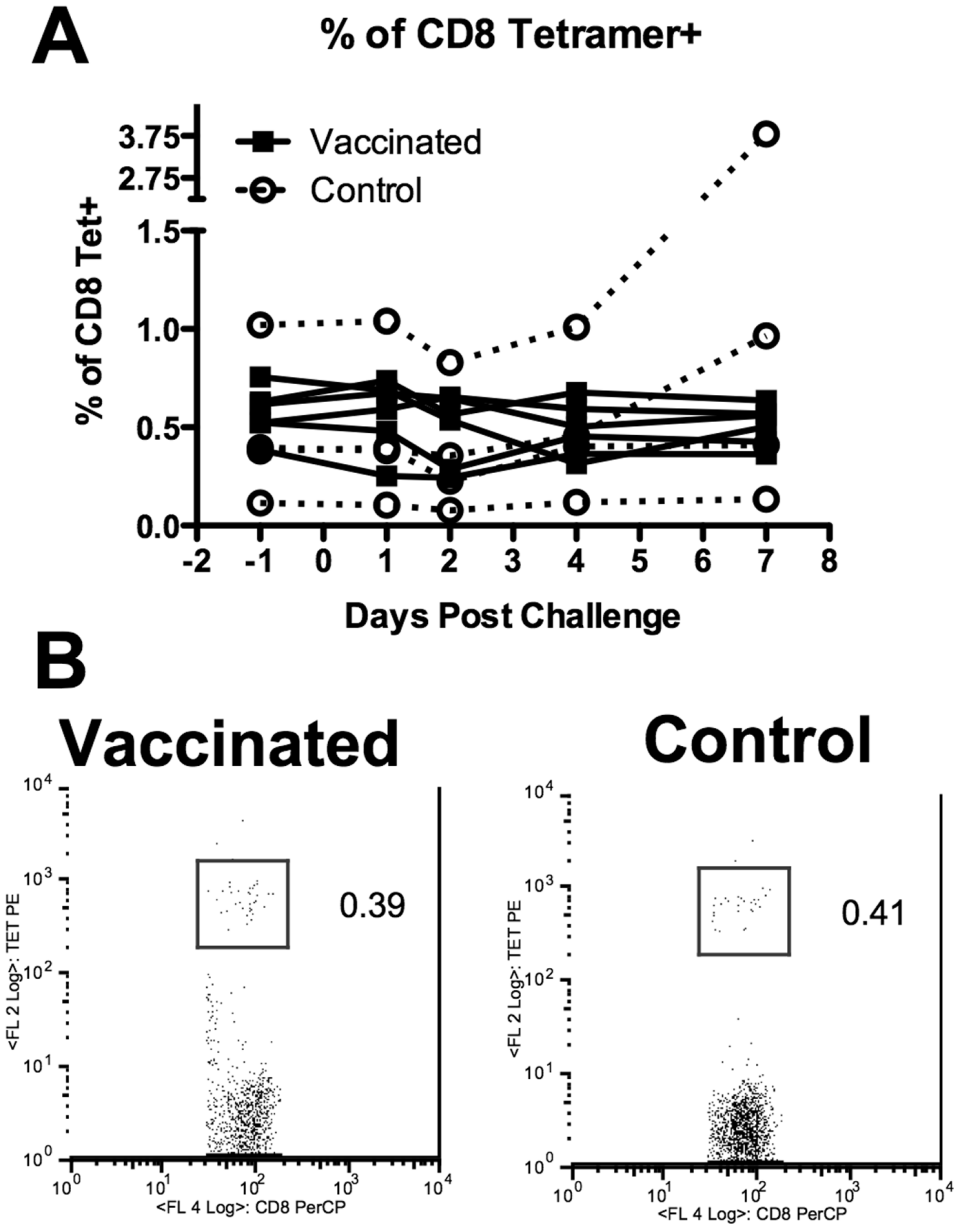
Frequency of tetramer positive cells is similar between vaccinated and control volunteers. Data shows percentage of CD8 cells within a CD8 gate with vaccinated as closed squares and control open circles. B) Representative FACS profile of one vaccinated and one control donor at day −1, one day before challenge with influenza virus.

Initially we analysed the percentage and absolute numbers of tetramer positive CD8^+^ T cells for each HLA-A*02 volunteer by FACS on the day before and up to day 7 post challenge. Surprisingly we found that the percentage of M1_58–66_ specific cells, shown in Figure 2A, was not different between vaccinated and control donors. A representative flow cytometry profile from one vaccinated and one control donor is shown in figure 2B, which shows cells gated on CD8 and the percentage of tetramer positive cells within that gate. The absolute number of M1_58–66_ antigen specific CD8^+^ T cells was also calculated using CD8 counts from the Trucount and these were not significantly different between groups (data not shown). Overall T cell responses to overlapping peptides spanning the entire H3N2 proteome [5] were tested and no significant differences were observed between vaccinated and control groups to most proteins, despite raised responses to NP in the vaccinated group before infection (figure S1) and [16], [17]. There is an overall trend of elevation of T cell responses in both groups 7 days after the challenge, which could mainly be CD4 dependent responses as described by Wilkinson et al., [21].

**Figure 3:**
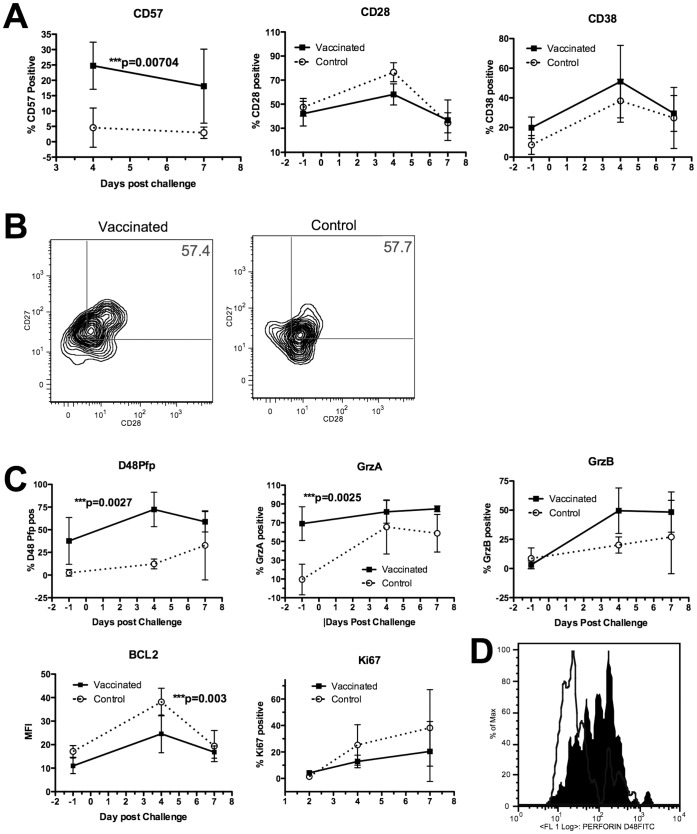
Surface and intracellular activation markers are enhanced on tetramer labeled cells from vaccinated donors compared to control. A) Time course between day −1 and 7 and expression of noted markers on M1_58–66_ tetramer labeled cells. B) Representative flow cytometry plot of M1_58–66_ tetramer positive cells labeled for CD27 and CD28 on day −1 showing similar profiles. C) Graphs plot the percentage of tetramer+ cells or MFI of tetramer+ cells with the noted markers. D) Representative flow cytometry plot of two donors showing control (open plot) and vaccinated volunteer (filled histogram) labeled with anti-D48 Pfp on day 4. Groups were compared using repeated measures ANOVA.

Since the number or proportion of antigen specific cells was not different between the groups we then examined the cell surface and intracellular phenotype of the M1_58–66_ CD8^+^ T cells. We examined the expression of CD27, CD28, CD38 and HLA-DR on the surface of the cells that are markers associated with activation and differentiation [19]. We found that the expression of CD27, CD28, CD38 and HLA-DR were not different between vaccinated and control donors by repeated measures ANOVA (figure 3A). The levels of CD57, which is a marker associated with either senescence or activation were different by repeated measures ANOVA (p = **0.00705) and CD57 was enhanced on cells from vaccinated volunteers. Double CD27^+^ CD28^+^ positive cells were not different between the groups and a representative flow cytometry profile is shown in figure 3B.

Further to the cell surface molecules we examined the intracellular molecules, granzyme A, granzyme B and perforin on M1_58–66_ specific cells; which are all associated with better levels of cytolytic activity. Significant changes in perforin expression were only detected using the more sensitive mab D48 that detects newly synthesized perforin [22] and we found that the perforin levels were higher in vaccinated volunteers by repeated measures ANOVA (p = **0.0027) (figure 3C). We found that granzyme A was elevated in vaccinated volunteers (repeated measures ANOVA p = **0.0025). Granzyme B was not different (figure 3C). These data imply that the vaccinated donors possess antigen specific cells that have developed the potential to be more cytolytic and this would then potentially correlate with faster virus clearance. In donors vaccinated followed by influenza challenge, levels of B Cell lymphoma-2 (BCL_2_) protein were reduced (repeated measures ANOVA p = ***0.00314) as shown in figure 2C. These changes may indicate more differentiated cells or cells that are more likely to apoptose. Figure 3D shows a flow cytometry profile from one vaccinated and one control donor showing increased perforin (D48 clone) in tetramer positive cells from a vaccinated donor at day 4.

**Figure 4:**
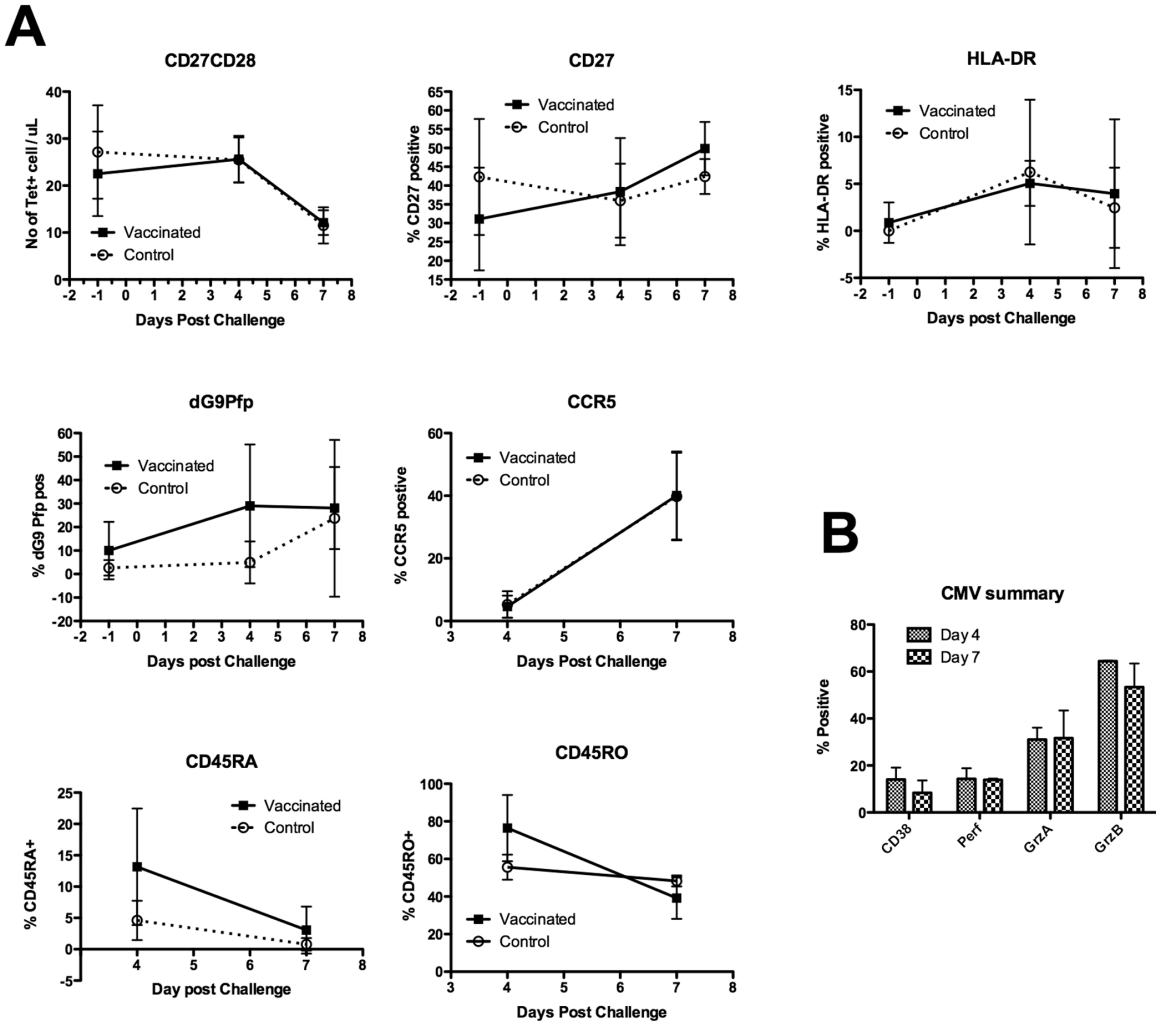
Analysis of markers that are not different between vaccinated and control volunteers and analysis of CMV specific T cells after influenza challenge. A) Cells were labeled for flow cytometry and percentage positive calculated using FlowJo. All donors are shown in the figures and indicate positivity for various markers after vaccination and or challenge with influenza virus. All groups were compared using repeated measures ANOVA. B) Analysis of phenotypic markers on CMV tetramer positive CD8 T cells showing different marker expression on these antigen specific cells.

Shown in figure 4A is the phenotype data that we obtained from the vaccinated/control and then influenza infected volunteers examining different surface and intracellular markers. Overall there are a number of proteins that show trends of difference that are similar to the statistically different changes shown in earlier figures, illustrating an overall picture that the M1_58–66_ specific CD8^+^ T cells are more responsive from the vaccinated volunteers than those from the control volunteers. Analysis of total CD4^+^ or CD8^+^ T cell populations could be useful but because of the unknown specificity of these cells we considered that it was better to measure either influenza or CMV antigen specific cells.

We also examined the phenotype of CMV specific cells in two vaccinated volunteers that were positive for the CMV tetramer and found that these did not change during the influenza infection (Figure 4B). This indicated that there was no bystander activation of CMV specific T cells during the challenge and that it is unlikely that the adjuvant effects of MVA are causing these changes in phenotype of M1_58–66_ specific cells.

This phase IIa study of a novel influenza vaccine was designed to test whether stimulating NP+M1 T cell responses were able to protect vaccinees against influenza [16], but here we particularly concentrate on changes in surface phenotype of the antigen specific cells after vaccination and influenza challenge. The phenotype of the influenza specific T cells was altered and these changes or enhancements of T cell phenotype have been found in other studies to be associated with protection against influenza [11]. Miller et al., found that antigen specific T cells were stimulated in a study of yellow fever and vaccinia vaccination and that vaccinated donors had enhanced activation profiles [23]. In other studies enhancement of perforin expression has been demonstrated on IFN-γ secreting cells after influenza vaccination [24]. We also find that perforin level is increased on antigen specific cells identified using tetramers after vaccination, which gives more insight into the response of antigen specific T cells after vaccination and challenge in humans.

The M1 specific CD8^+^ T cells have been shown to be protective in A2 transgenic mice [8], and HLA A2 positive donors commonly have detectable M1_58–66_ specific CD8 T cells [9]. Terajima et al., and Tu et al., [25], [26] show the presence of influenza specific T cells in human samples that have the potential to protect against novel strains of influenza. McMichael has shown association between cytotoxic activity of T cells and reduced virus shedding in humans [7]. Hikono et al., have shown that certain activated memory T cells can protect against subsequent influenza challenge [11] and that activation phenotypes may be more important than absolute numbers of memory cells. Murine antigen specific CD8^+^ T cells of a single specificity can be protective against influenza challenge after adoptive transfer [10] or by priming with a known peptide [8]. In humans protection against influenza is likely to involve different specificities of CD4^+^ and CD8^+^ T cells since it is likely that most adults will have previously been exposed to influenza. These studies could be extended in future to examine different specificities and HLA alleles. Another observation is that there are differences in T cell phenotype when the ELISPOT responses are similar. Despite the usefulness of the ELISPOT assay and its widespread use in trials of this type, it only gives one view of the T cell response and we recommend a more detailed T cell phenotype analysis to provide a more complete description of the effects of vaccination.

In conclusion, we have found that vaccination with an MVA construct containing NP+M1 in healthy volunteers led to more activated antigen specific CD8^+^ T cells and these cells have the potential to be more active in clearing virus because of higher levels of perforin and granzyme proteins. The approach of using tetramers in combination with phenotypic markers may also be a useful method to assess the immunogenicity of different vaccines.

## Supporting Information

Figure S1 **ELISPOT responses using peptides from individual genes Indicate rise in NP response after vaccination and infection and rise of H3 HA response after challenge.** IFN-γ ELISPOT assays were done using standard methods using overlapping peptides from H3N2 and other strains of influenza viruses.
(TIFF)

Table S1 **Summary of repeated measures ANOVA p values of marker analysis on M1_58–66_ specific CD8 T cells after vaccination and/or challenge.**
(DOC)
