# Supplementary material for: Examination of Influenza Specific T Cell Responses after Influenza Virus Challenge in Individuals Vaccinated with MVA-NP+M1 Vaccine
Source: PLoS One. 2013 May 3;8(5):e62778. doi: 10.1371/journal.pone.0062778 (PMC3643913; doi:10.1371/journal.pone.0062778)
Supplement: Table S1 — Summary of repeated measures ANOVA p values of marker analysis on M158–66 specific CD8 T cells after vaccination and/or challenge. (DOC) [file pone.0062778.s002.doc]

Supplementary Table 1. Summary of repeated measures ANOVA p values of marker analysis on M1_58-66_ specific CD8 T cells after vaccination and/or challenge.

| Marker^a^ | Vaccine^b^ | Time^c^ | Interaction^d^ |
| --- | --- | --- | --- |
| CD57 | 0.00704 | 0.085 | 0.333 |
| CD28 | 0.166 | 1.33e-05 | 0.0947 |
| CD38 | 0.252 | 0.00015 | 0.632 |
| Pfp D48 | 0.00275 | 0.00870 | 0.126 |
| GrzA | 0.00250 | 0.000201 | 0.00879 |
| GrzB | 0.0971 | 5.13e-05 | 0.0343 |
| BCL2 | 0.00313 | 1.34e-05 | 0.113 |
| Ki67 | 0.230 | 0.0039 | 0.265 |
| CD27/28 | 0.618 | 0.0001 | 0.592 |
| CD27 | 0.942 | 0.0249 | 0.0868 |
| HLA-DR | 0.833 | 0.106 | 0.824 |
| Pfp dG9 | 0.254 | 0.0143 | 0.233 |
| CCR5 | 0.970 | 5.88e-5 | 0.918 |
| CD45RA | 0.0943 | 0.0122 | 0.228 |
| CD45RO | 0.389 | 0.00176 | 0.0288 |

^a^Analysis of markers examined on antigen specific T cells after vaccination with an NP+M1 vaccine or control donors followed by challenge with intranasal influenza vaccine.

^b^p value with respect to variation caused by vaccine.

^c^P values with respect to variation attributable to time.

^d^p of both time and vaccine together.
